# Propagation Methods for the Conservation and Preservation of the Endangered Whorled Sunflower (*Helianthus verticillatus*)

**DOI:** 10.3390/plants10081565

**Published:** 2021-07-30

**Authors:** Robert N. Trigiano, Sarah L. Boggess, Christopher R Wyman, Denita Hadziabdic, Sandra Wilson

**Affiliations:** 1 Department of Entomology and Plant Pathology, Institute of Agriculture, University of Tennessee, Knoxville, TN 37996-4560, USA; sbogges1@vols.utk.edu (S.L.B.); dhadziab@utk.edu (D.H.); 2 Department of Plant Sciences, Institute of Agriculture, University of Tennessee, Knoxville, TN 37996-4561, USA; cwyman@vols.utk.edu; 3 Department of Environmental Horticulture, University of Florida, Gainesville, FL 32611-0675, USA; sbwilson@ufl.edu

**Keywords:** adventitious rooting, asexual propagation, compatibility, rooted cuttings, seed viability, whorled sunflower

## Abstract

*Helianthus verticillatus* Small, the whorled sunflower, is a perennial species only found at a few sites in the southeastern United States and was declared federally (USA) endangered in 2014. The species spreads locally via rhizomes and can produce copious seeds when sexually compatible genotypes are present. Vegetative propagation of the species via cuttings and the optimum conditions for seed germination have not been determined. To investigate asexual propagation via cuttings, stem sections were harvested in late May, June, and July in Knoxville, Tennessee (USA) and trimmed to a minimum of two nodes. The base of the cuttings was treated with either auxin or water, and grown in a Promix BX potting medium with intermittent mist and 50% shade for one month. Seeds were harvested from a population of multiple genotypes in Maryville, Tennessee and used to determine viability and the range of temperatures suitable for germination. A clonal population was developed and used for three years to assess sexual compatibility at three locations in Knoxville, Tennessee. Ninety-five percent of the cuttings from May rooted in two-to-three weeks and formed more than 20 adventitious roots per cutting with auxin and 18 with water treatments. The ability of cuttings to root decreased in June to about 20%, and none rooted in July with either water or auxin pretreatments. Pre-germination tetrazolium tests indicated that about 91% of seeds (achenes) were viable. Subsequent germination tests revealed high germination at varying temperatures (96 to 99% of seeds (achenes) germinated at 22/11, 27/15, and 29/19 °C), whereas germination was significantly inhibited by 33/24 °C. Fifty percent of the seeds germinated at 22/11 °C in 7.5 days, whereas only 2.0 to 2.5 days were required for 50% germination at 27/15, 29/19, and 33/24 °C. Seeds were not produced at any of the clonal planting locations during the three years. Vegetative propagation via rooted cuttings was successful in the mid-spring, seed germination was possible over a wide range of temperatures, and self-incompatibility was evident in this species. The results of this study will permit fast and efficient propagation of multiple and selected genotypes for conservation, commerce, and breeding of elite cultivars with disease resistance or other desirable attributes.

## 1. Introduction

**Figure 1:**
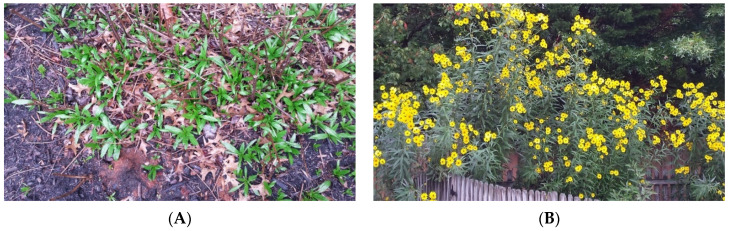
Growing habit of *Helianthus verticillatus*. (**A**) Basal rosettes of *H. verticillatus* in February. The dried stalks are from the previous year. (**B**) Flowering on tall stalks of *H. verticillatus* in late September when the plant produces attractive, yellow inflorescences.

*Helianthus verticillatus* or whorled sunflower is a perennial, herbaceous species restricted to a few prairie-like remnants and wetland sites in the southern United States (USA). These sites, with a few to several hundred plants each, are located in Georgia, Tennessee, Mississippi and Alabama [1,2], and a recently discovered small population in Virginia (Edward Schilling, personal communication). Because of the paucity of populations of *H. verticillatus*, it was designated as an endangered species in 2014 [3]. This sunflower species typically has three or four leaves at a node (oppositely arranged), and hence the designation “whorled sunflower”. This phyllotaxy distinguishes it from other similar and related sunflower species including *H. angustifolius* (narrow-leaved or swamp sunflower), *H. divaricatus* (woodland sunflower) and *H. microcephalus* (small-headed sunflower) [1,4,5]; see Edwards et al. [5] for a more complete description of *H. verticillatus*. *Helianthus verticillatus* is a self-incompatible species [6,7] that spreads locally via elongated, subterranean slender rhizomes, and thickened roots. The species produces copious amounts of small, black, or gray achenes or seeds [1] when compatible genotypes are present. Plants emerge in Tennessee (USA) as a basal rosette in February (Figure 1A) and grow to 3–4 m by August.

*Helianthus verticillatus* flowers profusely in September and through to mid-October or until a killing frost (Figure 1B). The plants have numerous 4–6 cm diameter inflorescences at the 20–30 cm of the termini of stalks with yellow ray flowers (petals) and green, turning brown to black, floral discs at maturity. The showy flowers attract over 30 species of potential pollinators, including many native bees and flies, and a variety of moths and butterflies [8]. The “eye-catching” floral display and the diversity of potential pollinators make this species a good candidate as an ornamental plant for the home garden [5]. 

Because *H. verticillatus* is reported to be self-incompatible [6,7], seed-derived plants will each have a unique genotype, and the unique characteristics of the parent may not be maintained by ordinary seed or sexual reproduction practices from generation to generation. Obligate outcrossing in this species probably maintains the moderate genetic diversity discovered in a study of the Georgia populations, suggesting that the majority of variation was individual and plant-based [5]. Therefore, because of obligate outcrossing nature of *H. verticillatus*, asexual methods of clonally reproducing and preserving elite or desirable individuals in the population will need to be developed. Clonal reproduction methods include various forms of in vitro culture or tissue culture, of which axillary bud proliferation or multiplication is the most common [9]. Tissue culture or in vitro methods have been applied successfully to the production of *H. annuus* (common sunflower) plants e.g., [10] as well as to *H. verticillatus* [11]. Typically, axillary bud proliferation [9] involves excising quiescent axillary buds and a small segment of the stem or a node (together, termed an explant) from an elite plant to be cloned. The explant is surface sterilized and transferred to a mineral and sugar-rich agar medium containing various growth regulators, including cytokinins and auxins. Elongated shoots are transferred to a medium containing auxin(s), and adventitious roots are formed. Plants are acclimated to a growing environment outside of the culture vessels, following established protocols. Tissue culture methods of producing clonal plants are often long, challenging, expensive, and labor-intensive. Furthermore, detailed protocols must be designed specifically for the species or genotypes of interest [9].

Another method of producing clones of economically important and elite selections of plants is by cuttings that can develop adventitious roots. This technique, which is a standard practice in the horticultural industries [12,13,14,15], has been applied to only a few sunflower species, including *H. debilis* Nutt ‘Flora Sun’ (Beach sunflower) [16], *H. angustifolius* (Swamp sunflower) (S. B. Wilson, unpublished data), *H. annuus* [17], and *H. tuberosum* (Jerusalem artichoke) [18]. Typically, the cut basal ends of young apical stems are treated with an auxin, e.g., indoleacetic acid (IAA), indolebutric acid (IBA), or naphthalene acetic acid (NAA), and are placed in a moist growing medium. The cuttings are grown with mist to lessen water loss [19] and in diminished light to stimulate adventitious root formation on the cuttings. Whole plants are acclimated easily and quickly to greenhouse conditions. The method is simple, easy to complete, appropriate for many genotypes, and relatively inexpensive compared to in vitro methods of production. 

Although some propagation methods exist for other closely related *Helianthus* species mentioned above, there is no information available concerning the vegetative or asexual propagation of *H. verticillatus* via rooted cuttings. This lack of information prevents the practical proliferation and conservation of multiple and individual genotypes, including elite germplasm. This absence of process for *H. verticillatus*, in turn, thwarts rigorous conservation efforts of multiple and diverse genotypes including directed breeding for the combination of desirable characteristics, such as stem color and resistance to powdery mildew [20]. Furthermore, commercial nursery production of plants was an integral component of the conservation and restoration program for the Tennessee coneflower (*Echinacea tenesseensis*), another endangered Asteraceae species [21]. Therefore, the primary objective of this research was to devise a clonal reproduction method for *H. verticillatus* by using adventitious rooting of stem cuttings, which would preserve other individual, as well as multiple, genotypes. Secondary thrusts were used to demonstrate conclusively sexual incompatibility of the whorled sunflower with a clonally propagated genotype, and to evaluate viability and conditions for germination of seed produced in a potential breeding program. Moreover, high seed viability and germinability are essential for the conservation and preservation of the genetic diversity within a population.

## 2. Results and Discussion

### 2.1. Rooted Cuttings

There are few reports of propagation of *Helianthus* species via rooted cuttings (e.g., [16,17,18]). Apical cuttings of *H. angustifolius*, having similar foliage, flower time, and form of *H. verticillatus*, had 100% rooting with or without auxin (Wilson and Campbell, unpublished data). In contrast, the commercial sunflower, *H. annuus*, which is also used as an ornamental plant, is typically propagated by open-pollinated seed [10]. Rooted cuttings (clonal reproduction) are used by plant propagators to maintain the uniformity of ornamental plants and to increase the number of elite or selected genotypes for production or breeding [22]. However, essential to the cutting method of propagation is the requirement to develop protocols for inducing adventitious shoots from the cuttings [23]. Therefore, one of our objectives of this study was to explore the time of the year to harvest stem cuttings for propagation and treatments that would induce adventitious roots.

**Figure 2:**
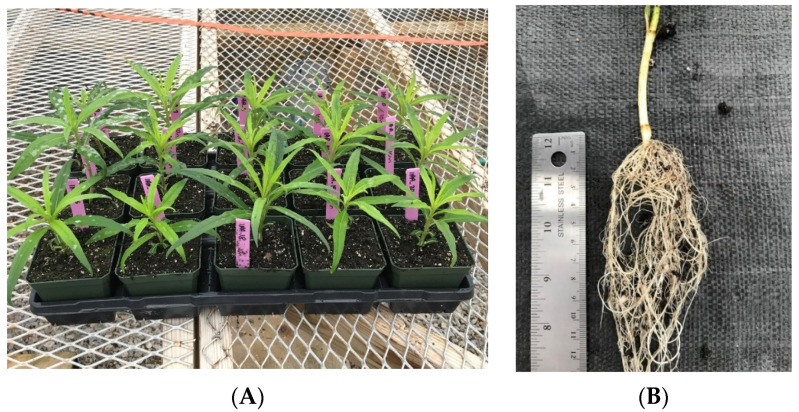
Stem cuttings and rooting of *Helianthus verticillatus*. (**A**) Stem cuttings *H. verticillatus* two weeks after placement in Promix BX. (**B**) More than 25 adventitious roots formed on a stem cutting of *H. verticillatus* after four weeks using shade and mist.

Cuttings from stems (Figure 2A) collected in late May and treated with IAA or water formed adventitious roots after two weeks from nodes that were covered with moist Promix BX soilless medium. After four weeks with mist and shade, stems treated with water or 250 ppm IAA solution for 5 min formed a mean number of roots of 18 (range 7 to >25) and 22 (range 3 to >25) roots/stem, respectively, and were statistically similar. In contrast, stems treated with IAA for 10 min formed a mean number of roots of 24 (range 12 to >25) roots per stem. Eighteen or 90% all stems across the three treatments had 25 or more adventitious roots/stem (Figure 2B). Although the mean number of roots/stem treated with the 5 and 10 min IAA treatments were statistically similar, plants treated with a 10 min IAA treatment had a significantly greater number of roots/stem than the stems treated with water only. Pretreatment of *H. debilis* Nutt. (‘Flora Sun’ or beach sunflower) cut stems with potassium salt of IAA (KIBA) (an auxin) formed more adventitious roots than no auxin pretreatment. However, the additional root mass did not affect the survivability of rooted cuttings after transfer to pots [16]. Our study also revealed an increased number of roots with the longer auxin treatment, but this did not affect the survivability of the rooted cuttings. In our study of cuttings of *H. verticillatus*, 95% of all stems formed adventitious roots regardless of pretreatment, were successfully transplanted to larger containers, and acclimated to greenhouse conditions. 

Typically, two axillary buds at a single node expanded during adventitious root development. The shoots of rooted cuttings grown in shade and mist conditions were very susceptible to powdery mildew caused by *Golovinomyces ambrosae* [20], but did not affect rooting or survivability of the cuttings. Plants were successfully overwintered in a bow house and each cutting produced 3–9 stems from rhizomes in the following spring (2018).

In stark contrast to the rooting experiment initiated in late May 2017 where 95% of the cuttings were rooted, only 20% of the cuttings collected in late June and pretreated with a solution of IAA for 10 min formed roots (mean of five roots/cutting). June-rooted cuttings failed to live after they were transferred to larger containers in the greenhouse. All cuttings made in late July failed to form adventitious roots regardless of pre-treatment with auxin. The timing of the acquisition of shoots to be rooted is critical. For example, successful rooting of *Stevia rebaudiana* Bertoni cuttings was dependent on the month shoots were harvested [24]. Similar significant seasonal effects on the ability of stem cuttings to produce adventitious roots have been described for many woody and herbaceous plant species, e.g., in [25,26,27]. 

Shoots of *H. verticillatus* exhibit extremely strong apical dominance (inhibition of lateral bud growth), and axillary buds at all nodes distal to the apical bud undergo very little development during the growing season. Only the upper 20–30 cm of the stem with axillary buds expands in the fall and converts to inflorescences. One possible explanation for the lack of axillary bud growth is that auxins (IAA) produced by the apical meristem and transported toward the roots (polar auxin transport) [28,29] inhibits expansion of the buds at nodes. Shoot tip excision permits axillary bud development through exhaustion of auxin in the stem subtending the bud [30]. In contrast to the auxin depletion explanation of apical dominance, some reports have shown that a lack of auxin is not correlated with the activation of axillary bud growth [31,32]. Fichtner et al. [33] convincingly showed that reallocation of sugars, especially trehalose 6-phosphate, to axillary buds permitted expansion of axillary buds of *Pisum sativum* following excision of the apical bud. It is probable that auxin, or auxin in combination with the reallocation of photosynthetic resources, are responsible for apical dominance. In *H. verticillatus,* apical dominance is apparently expressed by the production and polar transport of auxin down the stem. We hypothesize that auxin at the nodes in the May cuttings are sufficient to thwart axillary bud activation and growth, but are also sufficient to stimulate adventitious root formation. Sufficient concentration of endogenous auxin at nodes for adventitious root formation is evidenced in our study as rooting was obtained without the use of an exogenous source of auxin, which is necessary for cuttings of many horticultural species which are difficult to root [22,34]. Furthermore, two weeks after the cuttings were placed in a moist medium, one or more of the axillary buds at each node of the cutting resumed growth, supporting the contention that auxin concentration at the node had been depleted sufficiently to permit new apical buds to elongation. Axillary buds on the emerging stems were inhibited so that there was only one growing apex per new stem, indicating that the emerging shoots had restored apical dominance. Additionally, on the plants from which the cuttings were obtained for the rooted cutting experiment, axillary buds at the uppermost nodes resumed growth after about ten days (data not shown) and established apical dominance in each stem. Axillary buds subtending the new apical buds were quiescent until the fall when many became activated and produced flower stalks.

### 2.2. Clones for Incompatibility Study

**Figure 3:**
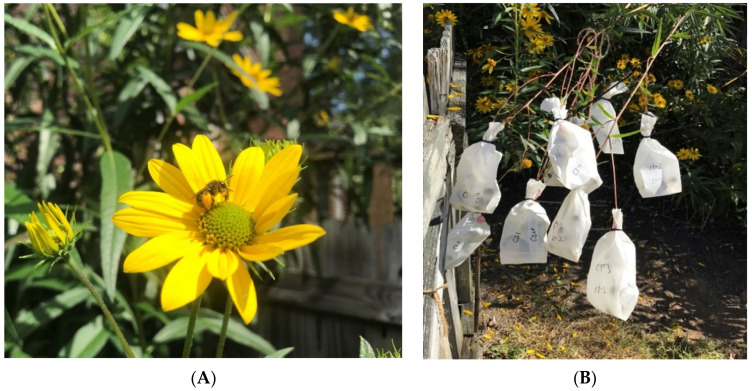
Floral visitor and seed collection for *Helianthus verticillatus*. (**A**) A native bee (*Melissodes* species; longhorn bee) visitor to an inflorescence of *H. verticillatus* in early-October 2018. Note yellow pollen on the leg of the bee. (**B**) Pollination bags covering multiple inflorescences after floral discs turned brown-black in mid-to-late October 2018.

All plants grew well and flowered prolifically in early-September to mid-October of all three years. Testing of plants with homozygous molecular markers for *H. verticillatus* [5,35] demonstrated genetic uniformity of the presumptive clones (Table 1). Potential pollinators were abundant and were similar to the type frequency reported for the Maryville, Tennessee site, except that honeybees were much more common visitors than described previously by Strange et al. [8]. Many of the native bee species carried observable pollen (Figure 3A). Honeybees as well as native bee species are efficient pollinators of the commercial sunflower [36,37,38]. Dissection of inflorescences within the pollination bags (Figure 3B) revealed that no black to gray achenes or seeds were formed in the three years of the experiment. We, therefore, concluded that *H. verticillatus* is self-incompatible and will not set seed unless a compatible genotype is present. Our experiments confirm the reports of self-incompatibility [6,7]. The determination of self-incompatibility is essential for breeding purposes. Use of homozygous markers, which are different for each breeding partner, quickly identifies heterozygous progeny (hybrids) and excludes any possibility of selfing in self-incompatible species such as the whorled sunflower. Horticulturally, a cultivar release of a specific *H. verticillatus* clone is more desirable than the release of multiple genotypes capable of abundant seeds, which can easily be spread by small birds, recruit new plants, and become weedy or invasive in gardens. 

### 2.3. Seed Viability and Germination Studies

**Figure 4:**
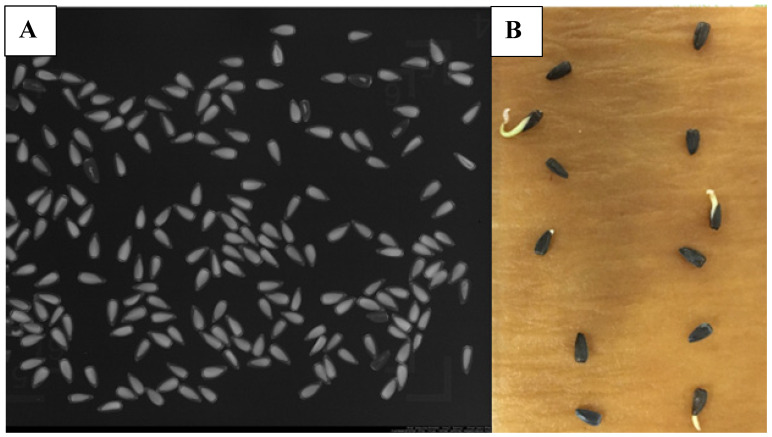
(**A**) Non-destructive X-ray analysis of *Helianthus verticillatus* seeds revealing presence (white) or absence (dark) of embryos. (**B**) Representative image of germinated (white-green, radicle emergence) seed.

Staining with tetrazolium (TZ) indicated the initial viability of a subsample of 12-week dry stored seeds to be 91%. This concurred with nondestructive X-ray analysis showing that 95% of the seeds were filled (contained a normal sized embryo) (Figure 4). Germination studies revealed that seeds display a high capacity to germinate quickly over a wide range of seasonal temperatures (Table 2). Final germination percent ranged from 75.5 to 98.8% among temperature treatments with greatest germination in simulated spring conditions (27/15 °C) and lowest germination in simulated summer conditions (33/24 °C). Average germination time ranged from 2.0 to 7.5 days, with germination occurring slowest at the coldest temperature (22/11 °C) compared to the other temperatures (Table 1). 

Post-germination TZ tests revealed that the majority of non-germinated seeds in the warmest treatment were viable. Lack of germination could be attributed to thermoinhibition of a small percentage (~20%) of seeds [22] or the fact that some seeds may not have met a physiological dormancy within the 28 days of the test [39]. While a number of other native and non-native *Helianthus* species are reported to have physiological dormancy [36], as well as physical dormancy [40], the ease of germination of *H. verticillatus* is consistent with that of the closely related *H. angustifolius* (swamp sunflower) where 19 of 26 accessions exhibited greater than 50% germination at 14 days [41]. 

The majority of *H. verticillatus* seeds in this study appeared to be non-dormant. While it is possible that seeds may have naturally overcome any existing dormancy due to an after-ripening effect (seeds were briefly dry stored before germinated tests could be conducted), freshly collected seeds were previously found to germinate on moist filter paper at 21 °C after one week (data not shown). Of further interest, seeds did not lose viability after seven months of dry and ambient temperature storage, suggesting that they are recalcitrant and have some tolerance to drying after seed development.

Unlike *H. verticillatus*, dormancy in *H. annuus* and other *Helianthus* species can be complex and involves many factors such as desiccation during seed formation, inhibitory action of the pericarp, and the seed coat, time, and environment for the full development of the embryo. Abscisic acid (ABA) levels generally are abated after several months of dry storage of *H. annuus* seeds [42] and references within and after nine months of dry storage, seeds were not dormant [43]. However, Inoka and Dahanayake [10] reported that *H. annuus* seeds required stratification for seed germination. 

High seed viability and the influence of temperature on germination of *H. verticillatus* were consistent with five other native *Helianthus* species and five genotypes of *H. annuus* reported by Castillo-Lorenzo et al. [40], reflecting their adaptability to different environmental conditions. While very warm temperatures (above 40 °C) have been found to be detrimental to the seedling development of *H. annuus* [43], *H. verticillatus* seeds were able to germinate at the highest temperature tested, but not at the same rate as seeds at the cooler temperatures. Even at the coldest temperature treatment, 50% of the seeds were able to germinate within 7–8 days consistent with seeds of high vigor. The ease of seed germination of *H. verticillatus* will be critical in future breeding efforts for powdery mildew resistance and novel flower morphology. 

## 3. Materials and Methods

### 3.1. Rooted Cuttings

The apical whorl of internodes and top six elongated internodes of 60 randomly selected plants from a mixed genotype population of *H. verticillatus* growing in Maryville, TN, USA were collected during the third week of May 2017. Two- or three-node cuttings were prepared from each stem, and the leaves from the bottom two nodes were removed. The base of 20 prepared cuttings for each treatment was placed randomly in either water or aqueous solutions of about 1400 µM (250 ppm) indole-3-acetic acid (IAA) for 5 or 10 min. The basal two nodes of each cutting were covered with moist Promix BX (Premier Tech, Rivière-du-Loup, Quebec, QC, Canada) contained in 10 cm plastic pots. Cuttings were placed in a mist chamber (12 s every 16 min) with 50% shade. The experiment was repeated in the third week of both June and July 2017. Percentage rooting and the number of adventitious roots per cutting was evaluated after one month for each date of cuttings, analyzed using an analysis of variance with the least square means calculated, and separated utilizing Tukey’s post hoc method [44] in R 3.3.1 (Vienna, Austria) [45]. Rooted cuttings from the May experiment were transferred to 8-L containers with Promix BX, grown under greenhouse conditions until September, and then transferred to a hoop house for overwintering. Stems emerging from the containers were counted in March of the following year (2018).

### 3.2. Clones for Incompatibility Study and Seed Production

In April 2018, plants (5–7 stems per pot) of one genotype propagated in 2017 were planted at each of three locations in Knoxville, TN, USA. In April 2018, young leaves from three different plants at each of the three locations were collected and genomic DNA extracted using the method described for *Viburnum farreri* [46]. To assure clonality of the plants, genomic DNA samples from *H. verticillatus* and *Cornus florida* L. (negative control) and distilled water (negative control) were amplified with eight homozygous *H. annuus* Simple Sequence Repeats (SSRs) [5,35] (Table 1) using the parameters described by Hamm et al. [46]. Amplification products were visualized using the QIAxcel ScreenGel version 1.6.0.10 [47], which can discriminate two bp [48]. Raw allele sizes were then statistically binned into allelic classes with FlexiBin an Excel macro [49].

Cloned plants at the three locations in Knoxville, TN, USA flowered in September/October in 2017 and 2018. After the inflorescence disks turned brown in mid-October, pollinator bags were placed over the stalks bearing numerous inflorescences, and the bags were affixed to stems with zip ties. Bags were collected in mid-November and seed production was assessed. The experiment was repeated in 2019 and 2020 with the following exception: more stems and flowers of the clone were present in each successive year. 

### 3.3. Seed Production, Viability and Germination

The multiple genotype collection of about 200 *H. verticillatus* plants in Maryville, TN (USA) was used for seed production via open pollination. Plants flowered in September and October of 2017, and about 15–20 inflorescences on 40 stalks were covered with pollinator bags (MIDCO Global, (Kirkwood, MO, USA) and affixed to the stem with zip ties. Achenes (seeds) were collected in mid-November, separated from somatic tissues, stored in glass vials at room temperature, and shipped via postal service to the University of Florida (Gainesville, FL, USA) in early February 2018. 

A subsample of seeds was subjected to non-destructive X-ray analysis using an Ultra Focus X-ray system (Faxitron Bioptics LCC, Tucson, AZ, USA) with embryo fill calculated using Faxitron Vision software. In addition, pre-germination seed viability was examined with 100 seeds using a TZ staining test adapted from the Association of Official Seeds Analysts (AOSA) [50] rules for Tetrazolium testing [51]. Seeds were cut laterally and stained overnight (18–24 h) at 37 °C in a 1% tetrazolium (2,3,5-triphenyl-2H-tetrazolium chloride) TZ solution. Seeds were considered viable when firm embryos stained evenly red (US Forest Service National Seed Laboratory, Dry Branch, GA, USA). 

In accordance with the Associate of Official Seed Analysts [50], individual treatments for all germination experiments consisted of four replications of 100 seeds. Seeds were surface sterilized with a 1.2% sodium hypochlorite solution for 10 min and then triple-rinsed with autoclaved (95 °C at 12PSI for 10 min) distilled water. Seeds that floated or had visible indication of pathogen or insect damage were discarded. Cleaned seeds were placed in 11 × 11 × 4-cm transparent polystyrene germination boxes (Hoffman Manufacturing, Albany, OR, USA) containing one sheet of germination paper on top of one sheet of blotter paper (Hoffman Manufacturing) moistened with 15 mL of autoclaved distilled water. Each of the four germination boxes containing 100 seeds were randomly placed into temperature, equipped with light-controlled incubators and cool-white fluorescent lamps (Percival Scientific, Model I30VL, Perry, IA, USA). Incubators were set to one of four alternating temperatures (22/11 °C, 27/17 °C, 29/19 °C, or 33/24 °C) with a 12-h photoperiod. The photosynthetic photon flux inside each chamber was an average of 81 µmol m^−2^ s^−1^ at empty shelf level. 

Germination of seed was monitored three times a week for a period of 28 d, and 2 mL of autoclaved distilled water was added as needed. A seed was counted as germinated when the radicle emerged out of the seed coat greater than 2.0 mm. At the end of the germination period, final germination percentage (FGP) and T50 (days to 50% of FGP) were determined per germination box. Any seeds that did not germinate by the end of the experiment were subjected to a post-germination viability test, as described above. Total seed viability was calculated as the sum of germinated seeds + dormant seeds.

Seed viability was assessed with 100 seeds using a tetrazolium staining method (Peters, 2007) over 18–24 h. Seeds with embryos that evenly stained dark pink to red were considered viable. Germination of seeds was evaluated using four replications of 100 seeds (50), each at the following four temperature regiments: 22/11 °C; 27/17 °C; 29/19 °C; and 33/24 °C within a 12-h photoperiod. Tests were conducted over 28 days. In addition, post-germination tetrazolium tests were performed on non-germinated seeds after 28 days to determine the percentage of dormant seeds.

For germination experiments, seeds were randomly assigned to 4 temperature treatments. Each temperature treatment consisted of 100 seeds pseudo-replicated four times within each chamber. Analysis of variance was performed in JMP Pro 15.0.0 (SAS Institute, Cary, NC, USA) to determine if there were significant germination differences among temperatures. When differences were significant, mean separation analysis was performed in JMP Pro 15.0.0 using the Tukey–Kramer honestly significant difference procedure at *p* ≤ 0.05. 

## 4. Conclusions

Stem cuttings harvested in mid-to-late May were easily rooted in moist medium, under shade and mist, and with or without auxin treatments. Cuttings made in June have diminished capacity to root and those collected in July will not produce adventitious roots. Apparently, polar transport of auxin from the apical meristem to the nodes is sufficient to stimulate adventitious root formation without the addition of exogenous auxin. Rooted cuttings of multiple genotypes are possible and are a suitable and simple method for mass propagation, and thus an excellent method to ensure conservation, restoration, and preservation of the whorled sunflower. *Helianthus verticillatus* is self-incompatible, as demonstrated by the lack of seed formation when multiple stems of a single genotype clone were present. Self-incompatibility is essential to a controlled breeding program and for the release of specific cultivars for home horticulture. Plantings of multiple sexually compatible genotypes produce bountiful seeds, which are essential to maintain and preserve genetic diversity in the population. Seeds of *H. verticillatus* are non-dormant and are capable of germinating readily after collection from plants. Seeds stored at room temperature and under dry conditions for three months also germinate readily under a wide range of temperatures, and do not lose viability after seven months and possibly longer under the similar storage conditions. The methods of propagation described herein are applicable to commercial horticulture, which is a complementary strategy for conservation and restoration of endangered species such as *H. verticillatus*. 

## Figures and Tables

**Table 1:** Primer codes, repeat motifs and primer sequences of simple sequence repeats (SSRs) used to determined clonality of *Helianthus verticillatus* plants at three locations in Knoxville, Tennessee, USA.

Primer Code	Repeat Motif	Primers: F = Forward; R = Reverse	Location One (bp)	Location Two (bp)	Location Three (bp)
BL006	(GTGA)_3_	F: CATGGGTGATCAATGGAGTG	264	263	263
(HV006)		R: CGGCACATAACAAGTGCTTC			
BL0012	(GTTA)_3_	F: CGAGACGGTTAAGAGCTTGC	337	337	337
(HV012)		R: GGTGTACAACCAACTCACACC			
BL0022)	(TAA)_4_	F: ACTTACCGTTGCATTTGGTG	107	107	107
(HV017)		R: TTATCCCTAGAACACGATTACAG			
BL0019	(GAAA)_3_	F: GAGTCCTGGCCTGAACAGAG	296	295	295
(HV026)		R: AAACTGCAATGTACCTTCTTGAC			
BL0024	(GTAA)_3_	F: CTCCCGCACTTCAAGCTAAC	125	124	124
(HV028)		R: CATACACCTTTGCGGTTTCC			
BL0031	(GAC)_4_	F: CCGGAAGATAACGACGAGTG	423	423	424
(HV031)		R: TCCATCGCTTTCCCTAAATC			
BL0042	(GGC)_4_	F: GGTTACAACGGTGGAAGTCG	363	363	364
(HV042)		R: TCCGGTTCACCAATTCATTC			
BL0048	(GAA)_4_	F: TTGTGGAGACGGTGAATGAG	221	220	220
(HV048)		R: TAACCGAACGACCATTCTTC			

BL Codes from Pashley et al. [35], doi: 10.1093/jhered/esl013. HV Codes from Edwards et al. [5].

**Table 2:** Average final germination percentage and number of days to 50% of final germination (T50) of *Helianthus verticillatus* seeds collected from Maryville, Tennessee (USA) on 2 November 2017. Seeds were germinated with light (12 h photoperiod) in germination boxes placed in incubators set at 22/11, 27/15, 29/19, and 33/24 °C for 28 days. Post-germination tetrazolium tests were conducted on all non-germinated seeds to determine percent dormancy (viability) ^1^.

Temp. (°C)	Germination (%)	T50 (d)	Dormant (%)
22/11	96.3 b	7.5 a	3.3 b
27/15	98.8 a	2.5 b	0.8 c
29/19	95.6 b	2.0 c	2.8 b
33/24	75.5 c	2.0 c	23.0 a

^1^ Means followed by the same letter are not significantly different according to the Tukey–Kramer honestly significant difference procedure at *p* ≤ 0.05.

## Data Availability

Not applicable.
